# Supplementary material for: Risk factors for sacrococcygeal pilonidal sinus: a systematic review and meta-analysis supplemented by genetic causal assessment
Source: Front Surg. 2026 Jan 7;12:1718589. doi: 10.3389/fsurg.2025.1718589 (PMC12819706; doi:10.3389/fsurg.2025.1718589)
Supplement: Supplementary file 2 [file Datasheet2.zip › Supplementary Data 2/MR_pipeline_p5e-8/forest_plots/ukb-d-1747_5.finngen_R12_L12_PILONIDALCYST.pdf]

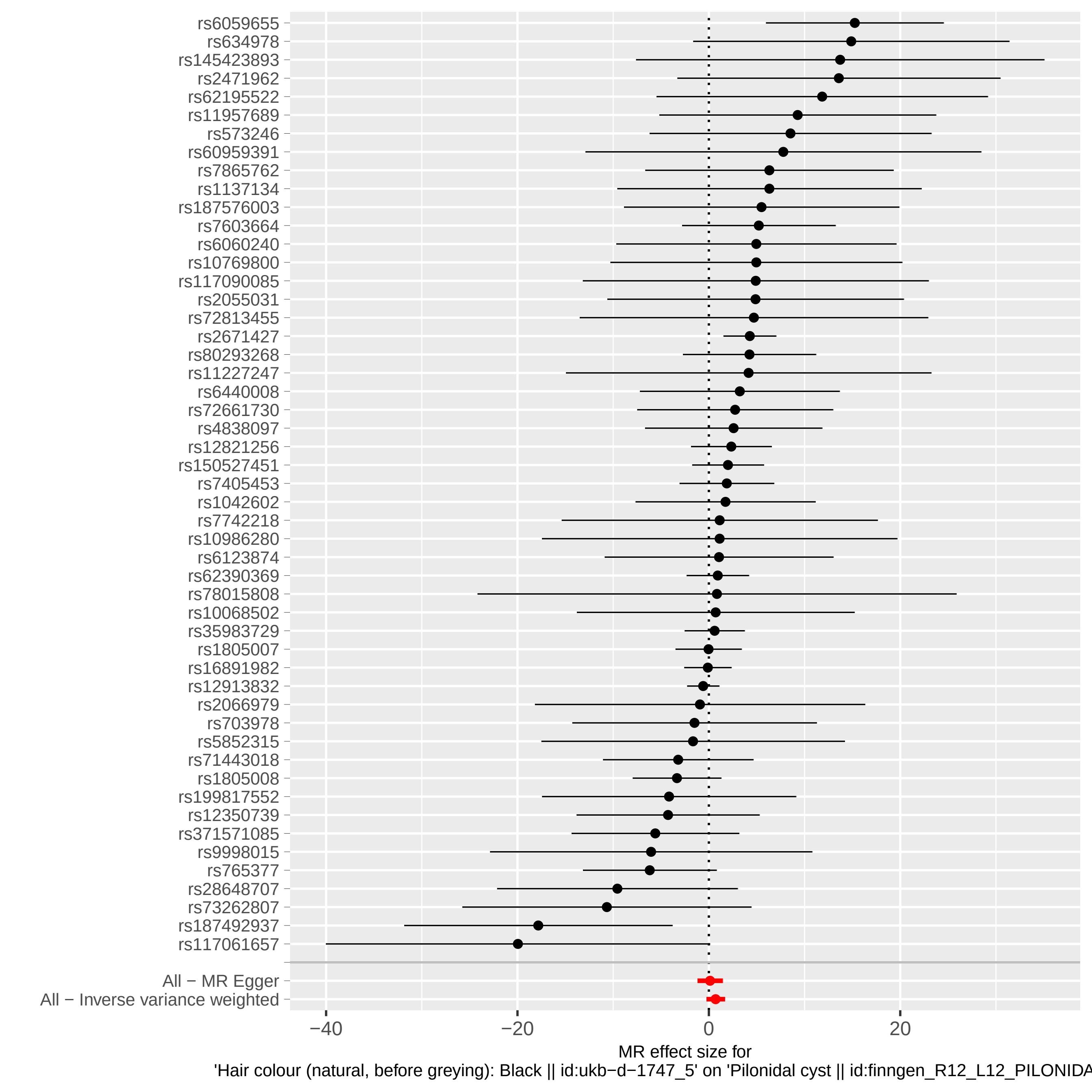
rs6059655
rs634978
rs145423893
rs2471962
rs62195522
rs11957689
rs573246
rs60959391
rs7865762
rs1137134
rs187576003
rs7603664
rs6060240
rs10769800
rs117090085
rs2055031
rs72813455
rs2671427
rs80293268
rs11227247
rs6440008
rs72661730
rs4838097
rs12821256
rs150527451
rs7405453
rs1042602
rs7742218
rs10986280
rs6123874
rs62390369
rs78015808
rs10068502
rs35983729
rs1805007
rs16891982
rs12913832
rs2066979
rs703978
rs5852315
rs71443018
rs1805008
rs199817552
rs12350739
rs371571085
rs9998015
rs765377
rs28648707
rs73262807
rs187492937
rs117061657
All − MR Egger
All − Inverse variance weighted
−40
−20
0
20
MR effect size for
'Hair colour (natural, before greying): Black || id:ukb−d−1747_5' on 'Pilonidal cyst || id:finngen_R12_L12_PILONIDA
